# Antimicrobials as Single and Combination Therapy for Colistin-Resistant *Pseudomonas aeruginosa* at a University Hospital in Thailand

**DOI:** 10.3390/antibiotics9080475

**Published:** 2020-08-03

**Authors:** Supanun Pungcharoenkijkul, Jantima Traipattanakul, Sudaluck Thunyaharn, Wichai Santimaleeworagun

**Affiliations:** 1 College of Pharmacotherapy Thailand, Nontaburi 11000, Thailand; supanun.pung@gmail.com; 2 Department of Pharmacist, Nopparat Rajathanee Hospital, Bangkok 10230, Thailand; 3 Division of Infectious Disease, Department of Medicine, Phramongkutklao Hospital, Bangkok 10400, Thailand; Jantima24Job@gmail.com; 4 Faculty of Medical Technology, Nakhonratchasima College, Nakhon Ratchasima 30000, Thailand; tanmicro@gmail.com; 5 Department of Pharmacy, Faculty of Pharmacy, Silpakorn University, Nakorn Pathom 73000, Thailand; 6 Pharmaceutical Initiative for Resistant Bacteria and Infectious Disease Working Group (PIRBIG), Nakorn Pathom 73000, Thailand

**Keywords:** biofilm formation, carbapenemase, *mcr-1*, minimal inhibitory concentration

## Abstract

Global infections with colistin-resistant *Pseudomonas aeruginosa* (CoR-PA) are increasing; there are currently very few studies focused on the antimicrobial susceptibility of CoR-PA isolates, and none from Thailand. Here, we investigated the impact of various antimicrobials, alone and in combination, via the in vitro testing of CoR-PA clinical isolates. Eighteen CoR-PA isolates were obtained from patients treated at Phramongkutklao Hospital from January 2010 through June 2019; these were classified into six different clonal types by using the enterobacterial repetitive intergenic consensus (ERIC)-PCR method, with a high prevalence of Group A (27.8%). The antimicrobial susceptibility was determined as the minimal inhibitory concentrations (MICs) using the epsilometer-test (E-test) method. The synergistic activities of six antimicrobial combinations were reported via the fractional-inhibitory-concentration index. All CoR-PA isolates were susceptible to amikacin, meropenem, and ceftolozane/tazobactam, but only 5.56% were susceptible to imipenem. In vitro synergistic activities were detected for amikacin with aztreonam, piperacillin/tazobactam, meropenem, and ceftazidime for 16.67%, 11.11%, 11.11%, and 5.55%, respectively. One CoR-PA isolate carried the *bla*_VIM_ metallo-β-lactamase gene; none carried *mcr-1* genes or detected plasmid-mediated AmpC β-lactamase or an overproduction of chromosomal AmpC β-lactamase. Seven CoR-PA isolates (38.89%) were capable of biofilm formation. In conclusion, CoR-PA isolates are highly susceptible to antimicrobials; the synergy observed in response to the various agents should be examined in a clinical setting.

## 1. Introduction

*Pseudomonas aeruginosa*, a nonfermentative Gram-negative rod-shaped bacterium, is a major cause of nosocomial infections, including those of the respiratory tract, skin, soft tissue, urinary tract, surgical sites, and the bloodstream. Infections are particularly prominent in patients with neutropenia and chronic lung diseases [1]. The global incidence of infections due to multidrug-resistant *P. aeruginosa* (MDR-PA) has been increasing; this includes *P. aeruginosa* strains that are resistant to carbapenem (CR-PA), which are particularly difficult to treat. The World Health Organization has listed CR-PA as one of the first-priority pathogens for the investigation and development of both new antibiotics and infection-control strategies [2]. In Thailand, 20% of *P. aeruginosa* specimens isolated from national hospitals from January to December 2019 were identified as CR-PA [3].

Infections with MDR-PA, including carbapenem-resistant strains, are associated with high rates of mortality, currently estimated at approximately 18–61% [1,4]; a combination antimicrobial treatment against CR-PA has been explored in cases that were resistant to treatment. However, the choice of monotherapy or combination therapy in cases of *P. aeruginosa* infection remains controversial. Previous studies revealed that the use of combination antimicrobial strategies (notably, those empirically designed), were more effective than monotherapy was for patients with *P. aeruginosa* infections; this was especially the case for critically ill and neutropenic patients. Additionally, several international consensus guidelines that addressed the optimal use of antimicrobial polymyxins recommended that invasive infections of CR-PA should be treated with combination therapy, regardless of the fact that there were no strong randomized control trials that proved the benefit of antimicrobial combinations in these settings [1,5,6].

Unfortunately, given the increasing use of colistin for the treatment of CR-PA infections, colistin-resistant (CoR) strains of *P. aeruginosa* have been reported. At this time, colistin-resistant *P. aeruginosa* (CoR-PA) has been documented only sporadically, but it will most likely become a serious problem in the near future. Data from South Korea, Iran, and Egypt documented the prevalence of CoR-PA at 7.4%, 14.9%, and 21.3%, respectively. Similarly, there are two recent publications that reported the emergence of CoR-PA in southern and central Thailand at rates of 1% and 1.6%, respectively [7,8,9,10,11].

At present, there are very few studies that focus on appropriate antimicrobial regimens to treat CoR-PA; various strategies, including the revival of older antibiotics such as aztreonam, remain attractive possibilities. In this study, we evaluated the in vitro susceptibilities of CoR-PA to various single antimicrobials and combinations of antimicrobials in order to optimize treatment. We also considered mechanisms associated with antimicrobial resistance and the prevalence of biofilm formations among the clinical isolates of CoR-PA.

## 2. Results

Eighteen clinical specimens of CoR-PA were isolated from various sites during a 10-year period. All CoR-PA strains were resistant to colistin, as shown using the broth-microdilution method (MIC > 16 µg/mL). All CoR-PA isolates were susceptible to amikacin, meropenem, and ceftolozane/tazobactam with minimal inhibitory concentrations (MICs) in the range of 0.75–16, 0.094–1.5, and 0.25–3 µg/mL, respectively. By contrast, only 5.56% of the CoR-PA isolates in our study were susceptible to imipenem, with MICs ranging between 1 and 6 µg/mL. Interestingly, 94.44% of the CoR-PA isolates were susceptible to aztreonam (breakpoint ≤ 8 µg/mL). The MIC range, MIC_50_, and MIC_90_ results, in response to piperacillin/tazobactam, ceftazidime, imipenem, meropenem, amikacin, gentamicin, ciprofloxacin, levofloxacin, ceftolozane/tazobactam, and aztreonam, are shown in Table 1.

An evaluation of the clonal relationships by using the enterobacterial repetitive intergenic consensus (ERIC)-PCR method revealed that the 18 isolates could be classified into six clonal types (A–F). Most strains were classified as clonal type A (*n* = 5), followed by clonal types B–D (*n* = 3) and clonal types E and F (*n* = 2), as shown in Table 2.

The in vitro synergistic activities of antimicrobial combinations, as determined with two E-test strips, are shown in Table 2 and Table 3. From the 18 CoR-PA isolates, the synergistic effects were detected for three isolates (16.67%); the most effective combination was amikacin with aztreonam, followed by amikacin with piperacillin/tazobactam and meropenem (11.11%), and amikacin with ceftazidime (5.55%). No synergistic responses were detected with combinations of amikacin with imipenem or amikacin with ceftolozane/tazobactam. Taken together, additive responses were identified when amikacin was supplemented with any one of the beta-lactam class of antibiotics (i.e., piperacillin/tazobactam, ceftazidime, aztreonam, ceftolozane/tazobactam, meropenem, or imipenem). No antagonistic effects were observed in response to any of the evaluated combinations.

We considered the possibility that the CoR-PA phenotype was associated with known resistance genes. One CoR-PA isolate (5.56%) produced metallo-β-lactamase (MBL) and carried the *bla*_VIM_ gene as determined by multiplex PCR; this CoR-PA isolate also tested positive on the E-test MBL strip (imipenem/imipenem + ethylene diamine tetra-acetic acid (EDTA): Liofilchem, Teramo, Italy), another finding that was indicative of metallo-β-lactamase production. Interestingly, none of the 18 CoR-PA strains carried a *mcr-1* gene. For phenotypic AmpC confirmation by cefoxitin-cloxacillin testing, plasmid-mediated AmpC β-lactamase or an overproduction of chromosomal AmpC β-lactamase were not detected among 18 CoR-PA strains (Table 4).

All CoR-PA isolates were evaluated for their capacity to produce biofilm formations using a microtiter-plate assay. The biofilm formation was classified into three groups according to the measured optical-density (OD) values; most strains were unable to generate biofilms (11 isolates or 61.11%). Of the seven isolates (38.89%) that were capable of biofilm formation, these were classified as moderate (five isolates) and weak (two isolates). All CoR-PA strains in clonal types B and E were capable of biofilm formation, and were classified as moderate and weak, respectively.

## 3. Discussion

The antimicrobial agent colistin is currently used only as a last resort for the treatment of multidrug-resistant (including carbapenem-resistant) *P. aeruginosa* infections, as it is well-known for having excellent bactericidal activity [12]. Unfortunately, colistin-resistant *P. aeruginosa* strains have been identified in various parts of the world, including Thailand; as such, it was critical to focus this study on CoR-PA bacterial strains. This is the first study in Thailand that explored in vitro antimicrobial susceptibilities, and the first study to ever examine the activities of specific antimicrobial combinations against CoR-PA strains. In this study, we identified colistin-resistant strains using the broth-microdilution method; in previous studies, this was typically performed using disk-diffusion, E-test, and agar-dilution methods [11,13,14,15,16].

Previous studies that featured in vitro antimicrobial activity against CoR-PA strains demonstrated a wide range of antimicrobial susceptibilities. For example, Abd El-Baky et al. [11] evaluated the in vitro antimicrobial susceptibility of CoR-PA strains isolated in Egypt; among their findings, most of the CoR-PA strains studied were resistant to cefepime, ceftazidime, and aztreonam, although 80% remained susceptible to meropenem, imipenem, piperacillin, and ciprofloxacin. In another study, Azimi et al. [16] found that all CoR-PA strains in their study were resistant to aztreonam, and most were resistant to other antimicrobial agents as well. However, they found that more than 90% of CoR-PA strains were susceptible to piperacillin/tazobactam, ciprofloxacin, and levofloxacin. These results can be compared to those presented in our study, in which we show that all CoR-PA strains isolated at our hospital in Thailand were susceptible to most of the evaluated antimicrobial agents.

Aztreonam was first identified in 1981; it is a synthetic monocyclic β-lactam antimicrobial agent that belongs to the family known as monobactams. Aztreonam has a broad spectrum of antimicrobial activity against Gram-negative aerobic bacteria, including *Enterobacteriaceae* and *P. aeruginosa*, but has no activity against Gram-positive or anaerobic bacteria. Earlier studies revealed that aztreonam is highly effective when used against Gram-negative bacteria, especially strains of *P. aeruginosa* [17,18,19]. Our results revealed that 94.44% of our CoR-PA isolates were susceptible to aztreonam with MICs of ≤ 8 µg/mL; only one strain was not susceptible, with an MIC of 12 µg/mL. These findings are similar to those previously reported by Mataseje et al. [15], and different to those reported by Abd El-Baky et al. [11] and Azimi et al. [11,16].

We also found that all CoR-PA strains were susceptible to ceftolozane/tazobactam (MIC range, MIC_50_, and MIC_90_ at 0.25–3, 0.75, and 1.5 µg/mL, respectively). This antibiotic formulation included a new cephalosporin and β-lactamase inhibitor combination and demonstrated strong activity against MDR strains of *P. aeruginosa* [20]. As such, as colistin resistance emerges, ceftolozane/tazobactam may be considered as a feasible treatment option for patients infected with CoR-PA. Testing of its efficacy in vivo is needed to confirm these findings.

To the best of our knowledge, this is the first study carried out in Thailand and globally that has identified in vitro synergistic activities in response to combinations of amikacin and aztreonam for CoR-PA strains; previous studies have examined the synergistic effects of aztreonam and amikacin against susceptible strains of *P. aeruginosa* [21,22]. Synergistic and additive effects associated with the combination of amikacin and aztreonam were observed in clonal types F and A of CoR-PA, respectively; as such, this combination may be among potential treatment options for CoR-PA infections, although studies that explore their in vivo efficacy are needed. Moreover, our study revealed an in vitro synergistic effect of amikacin and piperacillin/tazobactam against 11.11% of the CoR-PA strains. This degree of synergy was less than those identified in previous studies [23,24,25]; among the strains featured here, this antimicrobial combination primarily promoted additive effects with no observed antagonism. As aztreonam is currently unavailable in Thailand, the combination of amikacin and piperacillin/tazobactam is the best treatment available at this time for infections caused by CoR-PA strains; as noted above, further in vivo studies are needed.

Currently, the resistant mechanisms of colistin in *P. aeruginosa* are not entirely understood. Modifications or losses of the lipopolysaccharide (LPS) result in the reduction of colistin’s affinity to LPS. Additionally, the overexpression of efflux pumps, such as MexAB-OprM and MexXY-OprM, and a plasmid-mediated *mcr-1* gene, was reported in CoR-PA. However, none of the CoR-PA strains in our study carried the *mcr-1* gene. These results differed from previous reports in which CoR-PA strains were *mcr-1*-positive [11,14,26]. Moreover, we did not explore LPS modification or loss, or the overexpression of efflux pumps. Therefore, further study of the mechanisms of colistin resistance in our CoR-PA isolates is needed.

Surprisingly, we identified one CoR-PA isolate that carried the *bla*_VIM_ gene and produced metallo-β-lactamase; this strain was not susceptible to imipenem (MIC at 3 µg/mL), and was resistant to ceftazidime (MIC at 24 µg/mL), but was susceptible to piperacillin/tazobactam, ceftolozane/tazobactam, and meropenem, with MICs at 16, 0.38, and 1 µg/mL, respectively. These findings differed from those previously reported, in which all CoR-PA strains carried the NDM-1 gene and were reported to be resistant to carbapenem [15].

In this study, we found that 38.89% of the CoR-PA isolates were capable of forming biofilms. Biofilm-associated infections are typically difficult to treat and may require higher doses or combinations of antibiotics. Management of these patients might also include the removal of foreign material, especially in cases where device-related infections are suspected.

Our study has some limitations. First, we collected only 18 CoR-PA strains that were amassed over a 10-year period from an area of very low prevalence. Second, our study only evaluated in vitro susceptibilities; we did not examine optimal dosage regimens and clinical outcomes associated with CoR-PA infections in patients. Additional studies are required to more effectively assess the benefits of these novel treatments, and for the development of novel and efficacious antibiotics to be used to treat CoR-PA infections.

## 4. Materials and Methods

### 4.1. Bacterial Isolates

All CoR-PA strains were isolated from patients admitted to Phramongkutklao Hospital, a university hospital in Bangkok, Thailand between January 2010 and June 2019. The CoR-PA isolates were cultured and identified on the basis of their colony characteristics in blood agar; additional differentiation was performed using MacConkey agar (HiMedia, Mumbai, India) and cetrimide agar (Difco, Detroit, MI, USA), accompanied by standard biochemical testing. All CoR-PA strains had MICs of ≥ 4 µg/mL for colistin, as determined using a broth-microdilution kit (Compact Antimicrobial Susceptibility Panel; ComASP) according to the standards provided by the Clinical and Laboratory Standards Institute (CLSI), 2020 version [27].

### 4.2. Antimicrobial-Susceptibility Testing and Synergistic Activity

All clinical CoR-PA isolates were evaluated on their antimicrobial susceptibility by determining the minimal inhibitory concentration (MIC) values using E-test strips (Liofilchem, Teramo, Italy). The antimicrobials used in this study were piperacillin/tazobactam, ceftazidime, imipenem, meropenem, amikacin, gentamicin, ciprofloxacin, levofloxacin, ceftolozane/tazobactam, and aztreonam. The standard *P. aeruginosa* strain, ATCC 27853, was used as a control as per CLSI recommendations. The antimicrobial-susceptibility rates were determined from breakpoints of the MIC susceptibility for each antimicrobial agent according to the CLSI criteria [27]. This study also reported the MIC range, MIC_50_, and MIC_90_ of each antimicrobial agent for all isolates under evaluation.

The in vitro synergistic activities of specific antimicrobial combinations were determined by the E-test method. Briefly, the E-test consisted of placing two E-test strips on Mueller–Hinton agar (HiMedia, Mumbai, India) plates with bacteria spread at a 90° angle in cross-formation at the intersection between the MIC values determined for each antimicrobial agent. The six antimicrobial combinations were amikacin with one of the other agents under evaluation, namely, piperacillin/tazobactam, ceftazidime, ceftolozane/tazobactam, imipenem, meropenem, and aztreonam. The cumulative fractional-inhibitory-concentration index (ΣFICI) value of each combination was calculated. ΣFICI values calculated at ≤0.5, >0.5–1.0, >1.0–4.0, and >4.0 were interpreted as synergistic, additive, indifferent, and antagonistic effects, respectively. 

### 4.3. Determination of Clonal Relationships

The clonal relationships between the CoR-PA isolates featured in this study were determined using the ERIC-PCR method. Genomic DNA from all CoR-PA strains was extracted using a commercial kit (Thermo Fisher Scientific, Waltham, MA, USA). The PCR reaction mixture was as previously described [28,29], and used an ERIC forward primer 5’-ATGTAAGCTCCTGGGGATTCAC-3’, and ERIC reverse primer 5’-AAGTAAGTGACTGGGGTGAGCG-3’ for PCR amplification. Thermocycling was performed in a Biometra-TGradient Thermocycler (Biometra, Gottingen, Germany) as follows: 95 °C for 2.5 min, followed by 35 cycles of 46 °C for 30 s, 49 °C for 30 s, 72 °C for 3 min, and lastly, 72 °C for 10 min.

ERIC-PCR products were evaluated via 1.5% agarose-gel (Amresco, Solon, OH, USA) electrophoresis, and stained with ethidium bromide (Bio-Basic, Markham, Ontario, Canada). The classification groups used to differentiate between clonal types included patterns that differed with respect to three or more amplification products.

### 4.4. Detection of Metallo-β-Lactamase Genes and mcr-1

The PCR reaction mixture and primers used for the detection of metallo-β-lactamase genes were detected by multiplex PCR as previously described and with the primers listed in Table 5 [30]. PCR conditions were as follows: 94 °C for 3 min, 35 cycles of 94 °C for 30 s, 57 °C for 35 s, and 72 °C for 45 s, followed by a final elongation step at 72 °C for 5 min. The PCR reaction mixture and specific primers for *mcr-1* (305 bp) detection were as previously described [31,32], using *mcr-1*-forward primer 5’-CGGTCAGTCCGTTTGTTC-3’ and *mcr-1*-reverse primer 5’-CTTGGTCGGTCTGTAGGG-3’. PCR conditions were 94 °C for 3 min, followed by 35 cycles of 94 °C for 30 s, 53 °C for 35 s, 72 °C for 45 s, and a final elongation step at 72 °C for 5 min.

All PCR products were evaluated by 1% agarose-gel electrophoresis (Amresco) stained with ethidium bromide. Results were compared with amplicons of known metallo-β-lactamases and *mcr-1* genes that were used as positive controls.

### 4.5. Phenotypic AmpC Confirmation by Cefoxitin-Cloxacillin Testing

The cefoxitin-cloxacillin disk-diffusion test was performed to detect a chromosomal AmpC β-lactamase overproduction or presence of plasmid-mediated AmpC, using the cefoxitin-cloxacillin disk-diffusion test as previously described [33]. Briefly, cefoxitin (30 µg) disks were placed on Mueller–Hinton agar (HiMedia, Mumbai, India), and plates were inoculated with CoR-PA strains with and without 200 µg cloxacillin. The diameters of the cefoxitin-associated clearance zones were evaluated both in the presence and absence of cloxacillin. Differences in inhibition zones greater than 4 mm were considered to be positive for this detection.

### 4.6. Detection of Biofilm Formation by Microtiter-Plate Assay 

All CoR-PA isolates featured in this study were evaluated on their capacity to support biofilm formation using a microtiter-plate assay as previously described [34,35,36]. Briefly, CoR-PA isolates were adjusted to the 0.5 McFarland standard (1 × 10^8^ cfu/mL); 20 μL of CoR-PA isolates were grown in 96-well flat-bottomed sterile plates with 180 μL of Mueller–Hinton broth II (Difco, Detroit, MI, USA) supplemented with 1% glucose. Plates were incubated at 37 °C for 24 h. Wells were then rinsed with phosphate-buffered saline (PBS), and dry plates were fixed with 150 μL of methanol for 20 min and then stained with 0.1% Crystal Violet for 10–12 min. Plates were then washed with water, and the stain was solubilized in ethanol. Biofilm formation was evaluated as OD_550_ (optical-density at 550 nm) values determined by spectrophotometry. All experiments were performed in triplicate. The results of biofilm formation were interpreted as no, weak, moderate, or strong biofilm production as previously described by Kırmusaoğlu et al. [34].

### 4.7. Ethical Approval

This study was approved by the ethics review committee of the Institutional Review Board, Royal Thai Army Medical Department, Bangkok, Thailand (approval no. Q019b/62_Exp).

## 5. Conclusions

The results of our study indicated that clinical isolates of CoR-PA from our hospital were highly susceptible to numerous antimicrobial agents. We identified synergistic antimicrobial activity in response to amikacin combined with aztreonam, piperacillin/tazobactam, meropenem, and ceftazidime in studies performed in vitro. These combinations may emerge as potential treatment options for severe infections associated with infections with CoR-PA strains. Additional studies are needed to determine the overall efficacy and clinical outcomes. Several of these CoR-PA clinical isolates were also capable of forming biofilms. This point should be considered with respect to ongoing patient-management issues. In addition to antimicrobial therapy, it is important to consider the removal of foreign material if device-related infections are suspected.

## Figures and Tables

**Table 1:** Antimicrobial susceptibilities of colistin-resistant *P. aeruginosa*.

Antimicrobial Agents	MIC Range (µg/mL)	MIC_50_ (µg/mL)	MIC_90_ (µg/mL)	Susceptibility (%)
Piperacillin/tazobactam	1.5–32	6	16	88.89
Ceftazidime	0.25–24	1.5	4	94.44
Imipenem	1–6	3	6	5.56
Meropenem	0.094–1.5	0.25	1	100.00
Amikacin	0.75–16	1.5	4	100.00
Gentamicin	0.25–32	2	2	94.44
Ciprofloxacin	0.0625–8	0.125	4	83.33
Levofloxacin	0.38–2	0.75	1	88.89
Ceftolozane/tazobactam	0.25–3	0.75	1.5	100.00
Aztreonam	1–12	3	4	94.44
Colistin	>16	>16	>16	0

Abbreviations: MIC, minimal inhibitory concentration; MIC_50_, minimal inhibitory concentration required to inhibit the growth of 50% of the isolates; MIC_90_, minimal inhibitory concentration required to inhibit the growth of 90% of the isolates. Note: the antimicrobial susceptibility and MIC breakpoints are according to Clinical and Laboratory Standards Institute (CLSI), 2020.

**Table 2:** Clonal relationship and antimicrobial combinations of colistin-resistant *P. aeruginosa*.

Isolates	ERIC-PCR	AMK + PIP/TAZ		AMK + CAZ		AMK + IMP		AMK + MEM		AMK + C/T		AMK + ATM	
No.	Group	Activity	ΣFICI	Activity	ΣFICI	Activity	ΣFICI	Activity	ΣFICI	Activity	ΣFICI	Activity	ΣFICI
1	A	ADD	1	IND	1.33	IND	2	IND	1.41	IND	1.16	ADD	0.83
2	A	ADD	0.88	IND	1.41	IND	2	IND	1.15	ADD	0.71	ADD	1
3	A	IND	1.41	IND	1.41	IND	2	ADD	1	IND	2	ADD	0.62
4	A	ADD	0.62	ADD	0.66	IND	1.04	ADD	0.67	ADD	0.66	ADD	0.7
5	A	IND	1.16	ADD	1	ADD	1	ADD	0.58	ADD	1	ADD	0.7
6	B	ADD	0.88	IND	1.08	IND	2	IND	1.08	IND	1.5	ADD	0.84
7	B	SYN	0.42	SYN	0.44	ADD	0.58	ADD	0.58	ADD	0.54	ADD	0.83
8	B	ADD	0.63	IND	1.35	IND	2	IND	1.41	ADD	0.75	IND	1.5
9	C	ADD	0.87	ADD	0.7	IND	1.25	ADD	0.62	IND	1.25	ADD	0.7
10	C	SYN	0.35	ADD	0.75	ADD	0.75	ADD	0.59	ADD	0.87	SYN	0.5
11	C	ADD	0.62	ADD	0.88	IND	1.25	ADD	0.71	ADD	0.62	ADD	1
12	D	IND	1.25	ADD	1	IND	2	IND	1.16	IND	2	ADD	1
13	D	ADD	0.75	ADD	0.75	IND	1.66	IND	1.25	IND	1.25	IND	1.25
14	D	ADD	0.83	ADD	0.66	IND	1.66	IND	1.16	IND	1.32	IND	1.33
15	E	ADD	0.63	ADD	0.71	IND	2	IND	1.5	IND	2	IND	1.25
16	E	IND	2	IND	1.66	ADD	0.69	SYN	0.5	IND	2	IND	1.25
17	F	ADD	0.7	ADD	0.7	IND	1.08	IND	1.75	ADD	0.56	SYN	0.41
18	F	IND	1.33	ADD	0.83	ADD	0.7	SYN	0.33	ADD	0.88	SYN	0.42

Abbreviations: ERIC, enterobacterial repetitive intergenic consensus; PCR, polymerase chain reaction; AMK, amikacin; ATM, aztreonam; CAZ, ceftazidime; C/T, ceftolozane/tazobactam; IMP, imipenem; MEM, meropenem; PIP/TAZ, piperacillin/tazobactam; FICI, fractional-inhibitory-concentration index; SYN, synergistic; ADD, additive; IND, indifferent. Note: ΣFICI were interpreted as ≤ 0.5, synergistic; >0.5–1.0, additive; >1.0–4.0, indifferent; >4.0, antagonistic effect.

**Table 3:** Synergistic activity of the antimicrobial combinations of colistin-resistant *P. aeruginosa*.

Antimicrobial Combinations	Synergistic *n* (%)	Additive *n* (%)	Indifferent *n* (%)
Amikacin + piperacillin/tazobactam	2 (11.11)	11 (61.11)	5 (27.78)
Amikacin + ceftazidime	1 (5.55)	11 (61.11)	6 (33.33)
Amikacin + imipenem	0	5 (27.78)	13 (72.22)
Amikacin + meropenem	2 (11.11)	7 (38.89)	9 (50.00)
Amikacin + ceftolozane/tazobactam	0	9 (50.00)	9 (50.00)
Amikacin + aztreonam	3 (16.67)	10 (55.56)	5 (27.78)

Note: synergistic, additive, and indifferent were defined by the cumulative fractional-inhibitory-concentration index (ΣFICI).

**Table 4:** The clonal relationship, presence of metallo-β-lactamase genes and *mcr-1*, AmpC confirmation by cefoxitin-cloxacillin testing, and biofilm formation in colistin-resistant *P. aeruginosa*.

		Resistance Genes					
		Metallo-β-Lactamase					
Isolate No.	ERIC-PCR Group	IMP	VIM	NDM	*mcr-1*	AmpC Confirmation by Cefoxitin-Cloxacillin Testing	Biofilm Formation
1	A	X	X	X	X	X	No
2	A	X	X	X	X	X	No
3	A	X	X	X	X	X	No
4	A	X	X	X	X	X	No
5	A	X	X	X	X	X	No
6	B	X	X	X	X	X	Moderate
7	B	X	X	X	X	X	Moderate
8	B	X	X	X	X	X	Moderate
9	C	X	X	X	X	X	No
10	C	X	X	X	X	X	No
11	C	X	X	X	X	X	No
12	D	X	X	X	X	X	No
13	D	X	X	X	X	X	Moderate
14	D	X	X	X	X	X	Moderate
15	E	X	√	X	X	X	Weak
16	E	X	X	X	X	X	Weak
17	F	X	X	X	X	X	No
18	F	X	X	X	X	X	No

Abbreviations: ERIC, enterobacterial repetitive intergenic consensus; PCR, polymerase chain reaction. Notes: √, target gene present; X, target gene absent.

**Table 5:** Primer sequence used to identify metallo-β-lactamase genes.

Target Gene	Primer Sequence (5′-3′)		Amplicon Size (bp)
IMP	Forward	GGAATAGAGTGGCTTAAYTCTC	232
	Reverse	GGTTTAAYAAAACAACCAC	
VIM	Forward	GATGGTGTTTGGTCGCATA	390
	Reverse	CGAATGCGCAGCACCAG	
NDM	Forward	GGTTTGGCGATCTGGTTTTC	621
	Reverse	CGGAATGGCTCATCACGATC	
